# 

**Published:** 1887-01-01

**Authors:** 


### INDEX.

A

Abortion, incomplete....198
Abscess, mammary....116, 205, 426
Abscess, treatment of chronic....504
Academy of Medicine, New York....146
Acorn cocoa in infantile diarrhœa....117
Adonidin....206
Advertise. how to....125
Advice, take my....468
Aerial navigation....33
Agaricin for night sweats....425
Age. carboniferous....164
Ague....272
Air, purity of mid-Atlantic....77
Alabama, Surgical and Gynæcological Association of....37
Albumen test, Heller's....162
Albuminuria....32
Alligator's throat....384
Amenorrhœa....25, 26
American Medical Association....174, 302
Ammonium salicylate....152, 507
Ammonium, salicylate as an antipyretic...507
Amyloid, degeneration of kidney....265
Anæmia....250
Anæsthesia, dental....463
Anæsthetic, a new local....166, 426, 430, 517
Anæsthetic, Edison's....166
Anæsthetics....153
Anderson, Dr A. B....407
Andrews, Dr. Edmond....446
Aneurism, a case of femro-popliteal....57
101, 186.
Aneurism, cured by posit on....422
Anniston Medical Society....168
Ano fissure in....429
Anodyne, cutaneous....36
Anodyne for vesical irritation, an....369
Anthrax, perchloride in iron....161
Antifebrin....201, 203 206, 333, 465, 504, 510, 518
Antifebrin, a solvent for....510, 518
Antipyretics....469, 507, 509
Antipyrine....26, 110, 138, 157, 158, 59, 161, 212, 407, 470, 501, 510, 511
Antipyrine, an incompatible of....26
Antipyrine hypodermically as a substitute for morphine....510, 511
Antipyrine in bilious headache....470
Antipyrine in fever, arsenic and....110
Antipyrine in pneumonia....157
Antipyrine in puerperal fever....407
Antipyrine in typhoid fever....159
Antipyrine on the temperature in childr'n..158
Antipyrine, the proper use of....161
Anti rabies, inoculation. a new....164
Antiseptic dressing, latest Listers'....31
Antiseptic mouth wash....430
Antiseptic powder....518
Antisepsis, thorough and complete....460
Aphorism, surgical....366
Appendix, vermiformis. perforation of....392
Arago's measurement of steam....427
Arctic cold....516
Arseniate of Strychnia....19, 206
Arsenic and Antipyrine in fever....110
Arsenic and lithia for diabetes....342
Arsenic in wall paper....468
Artery, ligation of external illiac....'01, '86
Artery, ligation of Femoral....57
Artesian well at Galveston, the....428
Asbestos paper....252
Aseptic, dressing of fractured wounds....359
Association, American Medical....174, 302
Association of Alabama, Surgical and Gynecological....27
Association of Georgia, Medica ...38, 87, 126, 214
Association of Georgia. to the members of...87
Association, Ogeechee Medical....124
Association, Stone Mountain Medical...258, 345
Asthma, pyridine in....238
Atlanta, a dental school in....257
Atlanta Board of Health....125
Atlanta Medical College....168, 388
Atlanta Medical Society....38
Atlanta, our hospitals in....347
Atmosphere of caves....119
Atmosphere, resistance of....77
Atrophy, infantile....425
Atropine and carbolic acid....382
Atropine in hœmoptysis....432
Atropine in nocturnal earache of children 211
Avery, Dr. James....212

B.

Babies, water for....334
Baird, Dr. James B....184
Ballooning. high....428
Banchelle, Dr. L. B....492
Bandage, after delivery....37
Barbasco....32
Barium, chloride for vascular diseases....472
Barry, Dr. A....475
Bath, hot....68
Bathing, cold....244
Bath in fevers, salt....200
Bed wetting, red wine in....458
Bedside, observations at the....5
Bees, something new about....471
Belladonna in sterility of females....290
Benton, death of Dr. J. M....82
Berberis, Aquifolium....289
Bergeon's method of treating consumpt'n....330
Bernays. Dr. Augustus C....63
Berry, Dr. J. J....366
Bichloride of mercury as a germicide....490
Bicycles....124
Bill, the dissection....390
Biniodide of mercury in diphtheria and....80
Biniodide of mercury in scarlet fever....117
Bismuth as a dressing, subnitrate of....420
Bismuth in ulcerations, the subiodide of....241
Bismuth in sweating feet....324
Bisulphide of carbon....80
Black eye....161
Bladder, stone in....21
Blennorrhagia....123
Blood, sugar in the....251
Bloody flux....296
Board of Health, Atlanta....125
Bodies, removal of foreign....160

Body, how to preserve the dead....513
Boils by injection, the cure of....332
Boomerang, a....520
Boracic acid in leucorrhœa....377, 506
Borscheim, death of Dr E....432
Bouchelle, Dr. S. B....5
Brain, quantity of....261
Brain, surgery....370
Brain, tumors, localisation of....115
Bright's disease....76
Brochin, Dr. A....494
Bromide of ethyl....509
Bromide of ethyl with chloroform....112
Bronchitis....81, 165, 167, 297, 509 519
Bronton, Dr Daniel E....256
Boyce and the Virginia Medical College....391
Bucklin, Dr. C. A....450
Bullard, Dr. W. L....97
Bunting, Dr. Bose R....497
Burns....32, 76, 250
Burns, Tannin in....32
Buttermilk in sick stomach....205

C.

Cæsarian, operations, new german....319
Cæsarian, section on a cow....272
Caffiene and theine, action of....201
Calculi vesical....328
Calculus, renal....155
Calomel in diptheria....32
Camphor, charcoal and....281
Camphorated, Dover's....166
Cancer of the stomach....166
Cancers....285
Cannabin tannate....205
Cannabin indica in chronic dysentery....253
Carbolic acid, atropine and....382
Carbolic acid in felons....122
Carbolic acid on temperature, effect of....509
Carbon Bisulphide....80
Carboniferous age....164
Carbuncle, antiseptic treatment of....26
Carbuncle, carbolic and salicylic inject'ns..410
Caries, dental....519
Carminative, superior....35
Castration of criminals....514
Cataract extractions, a new era in....468
Cataract operation, after treatment of....246
283, 454
Cataract operations, two hundred....450
Catarrh, acute and chronic n sal....36
Catarrh, neurotic treatment of....424
Catarrh of the stomach, dietary in....412
Caution against septic germicides....1
Caves, atmosphere of....119
Cementation....120
Certificate, a death....168
Charbon, treated by excision....514
Charcoal and camphor....281
Charcoal, an antidote to strychnine poisoning....510
Charcot, Professor....524
Chenopodium, anthelminticum....323
Children, an enema for convulsions in....474
Children, colic in....35
Children, constipation in....298
Children, diarrhœa....341
Children, paralysis of....368
Children, spasmodic croup in....382
Children, threadworms in....20, 209, 343
Children, tonic for....81
Chills....255
Chionanthus, virginica in jaundice....36
Chisholm, Dr. J. J....112, 454
Chloroform....7, 112, 461
Chloroform and ether....461
Chloroform, bromide of ethyl with....112
Cholagogue pills....387
Cholera....24, 118, 385
Cholera infantum....342, 343 387
Cholera in hogs....17
Cholera morbus....253
Cholera, precautions against....118
Chorea....195, 431
Christopher, Dr. H....184, 230
Cimicifuga for headache....211
Circulation not essential to life....163
Circumcision, epilepsy cured by....147
City physicians, our....39
Clergyman's sore throat....243, 385
Cocaine cotton....254
Cocaine in dysentery....255
Cocaine inebriety....289
Cocaine in medicine, possible substitute....382
Cocaine in minor surgery....404
Cocaine internally....513
Cocaine, overdose of....199
Cocaine, physical action of....462
Cocaine poisoning....169, 289, 510
Cocaine, rival to....162
Cocaine, stenocarpine the new substitute for....467
Cocaine, toxical properties of....510
Cohen, Dr. J. Solis....312
Colds, treatment of....297
Colic, hepatic....32
Colic in children....35
Colic, infantile....81
College, Atlanta Medical....388
College, Southern Medical....37, 169, 388, 476
Collodion of iodoform....240
Commencement exercises, Southern Medical College....169
Compound fractures....155
Congestion, hepatic....473
Congress, International Medical....83, 256, 260,
344, 388, 390, 433, 434, 478
Conium, hydrobromate....205
Conjunctivitis....80, 385
Constipation habit....198, 387
Constipation of children....298
Constipation, simple....244
Consumption....299, 494, 473, 518
Consumption, gaseous enemata in....299
Consumption contagious, is pulmonary....494
Consumptives, soothing mixture for....473
Convulsions....139, 343, 386, 474
Convulsions of children, an enema for....474
Convulsions, puerperal....386
Copeland, Dr. W. P....369
Corkscrew, a pocket....208
Corn salve, a novel and painless....374
Corpulency, the treatment of....497
Cotton root as a uterine hæmostatic....211
Cough mixture....122, 210
Cough, quinine in whooping....74
Cough, whooping....64, 79, 165, 243
Courtesy, want of....86
Cow, cæsarian section on a....272
Crab, hermit....164
Cranium, fracture of....362
Creasote in Phthisis....462
Criminals, castration of....514
Croup....14, 204, 382, 416
Croup, antiseptic treatment of....204
Croup in children, spasmodic....382
Croup, prognosis of....14
Croup, turpentine in....416
Crystals of gold....340
Cupboard, the skeleton in the....472
Cutaneous anodyne....36
Cystitis, remedy in....85, 123
Cystotomy, suprapubic....71

D.

Death certificate....168
Decapitated animals, transfusion upon the 315
Decapitation, consciousness after....251
Delirium of typhoid fever....342
Delivery, bandage after....37
Dental anæsthesia....463
Dental caries, an anodyne for....519

Dental deparm'nt South'n Medical College.256
Dental school in Atlanta.....257
Dentition, painful.....123
Dercum, Dr Francis X.....443
Disinfectant, mixture for apartments.....210
Diabetes, arsenic and lithia for.....342, 519
Diabetes, Melitus.....296
Diabetes, a specific for.....381
Diarrhœa.....81, 117, 297, 341, 385, 386, 387, 423
Diarrhœa, chronic.....81
Diarrhœa, infantile.....117, 341, 518
Diarrhœa of children.....331
Diet for nervous exhaustion, the English system of.....61
Diet, the water.....25
Digestion of infants.....72
Dilation of uterus, new method.....197
Diphtheria.....32, 53, 80, 195, 290, 386, 430
Diphtheria, biniodide of mercury in.....80
Diphtheria, calomel in.....39
Diphtheria with rare qualifications,a case of..53
Diploma disgraced, a.....389
Discoveries, late.....34
Disease, the house fly as a distributor of.....311
Disease, barium chloride for vascular.....473
Dislocation of humerus.....426
Dissection bill, the.....390
Doctor's fee.....84
Dogs, military.....383
Dovers' camphorated.....166
Dovers' syrup.....385
Dramin.....25[illegible]
Duality of the brain.....261
Durham, Dr. A. F.....4
Dynamite.....354
Dysentery.....20, 249, 253, 255, 288, 296, 297, 375, 380 421, 463
Dysentery, epidemic.....90
Dysentery, etiology of.....249
Dysentery, paracoto in.....375
Dysentery, sarr cenia in sporadic.....288
Dysmenorrhœa, headache of.....81
Dyspepsia, flatulent.....474, 518

E

Ear, how to syringe the.....117
Ear, irrigating the.....389
Ear, secondary syphilis of middle.....268
Ear, suppurative inflammation of the.....282
Earache.....254, 341, 429
Earaches of children, nocturnal.....211
Earnest, Dr. J. G.....140
Earth, the living.....294
Earthquake shocks upon health.....104
Echinocæa for typhoid.....118
Eclectic, nullification by an.....257
Edison's anæsthetic.....166
Editor's son, death of managing.....83
Eggs, do not eat raw.....384
Electric lamp.....78
Electric magnet, extraction of an iron splinter from the eye by.....464
Electric motor, another.....33
Electrical ice cream poison.....515
Electricity, a form of matter.....428
Electricity, application of.....103, 162
Electricity in neuralgia, new method.....75
Electricity, lactation and.....373
Electricity, wonderful advance in.....252
Elephant, man the.....191
Elixir of iron, quinine, and strychnia pyro..79, 293
Embalming.....159
Enceinte patients. quinine in.....6
Enema for convulsions in children.....474
Enemata, gaseous.....299 312, 330
Enemata in diarrhœa, ice water.....115
Epidemic, insects as authors of.....120
Epilepsy, cured by circumcision.....147
Epilepsy, the cure of.....335
Epistaxis, treatment of grave.....379, 412
Equine, founder.....379
Ergot again.....511
Ergot in ophthalmic practice.....512
Erigeron, oil of.....457
Eruptions, chronic skin.....81
Eruptions, medicinal.....337
Eruptive fevers.....431
Ether, warm.....257
Ethyl, bromide of.....112, 509
Eucalyptol.....80, 377
Eucalyptol injections in tuberculosis.....377
Eucalyptus honey.....514
Eucalypt s in whooping cough and.....509
Europe, mortality in.....389
Erving, Dr. W. E.....142
Eye affections, iodol in.....292
Eye, Black.....161
Eye, extraction by an electro-magnet of an iron splinter from the.....464
Eye lotions.....287
Eye, proving the soundness of an.....207
Eyes, rest for the painful.....149
Exhaustion, English diet for nervous.....61
Extirpation of uterus, vaginal.....75

F.

Fatigue, professional.....207
Fees, doctors'.....84
Fehlings' solution; a substitute for.....31
Felons, carbolic acid in.....122
Femoral artery, ligation of.....57, 186
Femur, fractured.....70
Fever, bilious.....110
Fever, eruptive.....431
Fever Hay.....372
Fever, hemorragic malarial.....213, 239, 277, 291
Fever intermittent.....110, 290
Fever, malarial.....110
Fever, mixture.....121
Fever puerperal.....407
Fever, remittent bilious.....110
Fever, scarlet.....182
Fever, types.....521
Fever, typhoid.....21, 159 204, 342, 458, 513
Fever, typho-malarial.....390
Fevers, salt-baths in.....200
Fish story, scientific.....78
Fissure in ano.....429
Fœtus, maternal influence on.....190
Food, laxative.....226
Forceps, what about.....7, 201
Foreign bodies, removal of.....160
Forei n body in alimentary canal of an infant.....381
Fowler's solution for nervous condition of Menopause.....511
Fowler's solution, poisoning by.....324
Fracture of Cranium.....362
Fracture of patella.....162
Fracture compound.....155
Freire, Dr. Domingos.....252
Friends must write, our.....520
Furuncles, abortive treatment of.....418

G.

Galveston, artesian well at.....428
Gann, Dr.....124
Gas Lamp, incandescent.....339
Gas locomotion.....340
Gaseous. enemata.....299, 312, 330
Gaston, Dr. J. McF.....1, 57, 87, 101, 141, 177, 186, 263, 214, 392, 398, 523
Gaston's operation.....523
Gastrotomy fo removing a swallewed knife..63
Geese, wild.....383
Gelvin.....30
Genitals. pruritus of the female.....473
Georgia Medical Associat'n of..38, 87, 126, 174, 214
Georgia Medical Association, to the.....87
Germany, letter from.....66
Germicide, bichloride of mercury as a.....490
Germicides, caution against septic.....2

Ghosts, what of.....120
Gibier, Dr. Paul.....261
Glandular affections, ointment for.....122
Gleditschine.....475
Gleet.....29
Glycerine in phthisis.....298
Goldtsnoven, Dr. E. van.....270, 354
Goitre, parenchimatous.....425
Gold, crystals of.....340
Gonorrhœa.....27, 209, 419, 429
Gonorrhœa, chronic.....27
Goodell, Dr. William.....498
Graves' disease, an unusually rapid cas.....376
Gray, Dr. James A.....389, 432
Green, Dr. G. A.....53
Green, Dr. W. L.....475
Grothan, Dr. O.....359
Gynecological Association of Alabama.....37

H.

Hematemsis.....341
Hemorrhage.....122
Hemorrhage, pulmonary.....21
Hemorrhage, uterine.....165
Hemorrhoids.....74, 121, 167
Hemostatic, a new.....30
Hemostatic, uterine.....211
Hair follicles, inflammation of the.....165
Hair, hygiene of the.....414
Hair, removal of the superfluous.....156
Hair, pruritus vulvæ due to wild.....211
Hamilton, Dr. C.....272
Hamilton, Dr. J. L.....388
Hardon, Dr. Virgil O.....45
Harrison, Dr. J. F.....388
Hay fever.....373
Headache, bilious.....470
Headache, cimicifuga for.....210
Headache in pregnancy.....200
Headache, neuralgic.....367
Headache of dysmenorrhœa.....81
Headache, prescription for.....253
Headache, sick.....419
Health, Atlanta Board of.....125
Health, influence of earthquake shock.....104
Health of Los Angeles.....108
Heller's albumen test.....162
Hemirheumatism.....31
Hemorrhagic malarial fever.....213, 249, 277, 291
Henry, Dr. William.....362
Hepatic colic.....32
Hepatic congestion.....473
Hermit crab.....164
Hernia, a new operation for.....375
Hernia, cerebri.....362
Hernia, radical cure of.....227
Hernia, strangulated.....160
Herniotomy, laparatomy as an aid to.....22
Hobbs, Dr. A. G.....321, 486
Hog cholera.....17
Home journals, support your.....84
Hospital, Ivy Street.....37
Hospitals in Atlanta, our.....347, 389
House fly as a distributor of disease.....31
How to advertise.....125
How to preserve the dead body.....513
Humerus, dislocation.....426
Hunt, death of Dr. J. P.....299
Hydrocephalus, a twin child with.....141
Hydrophobia, inoculations, Pasteur's.....460
Hydroquinone.....506
Hypermetropic.....11
Hypnone.....288
Hypnotics, some new.....13, 508
Hypnotism.....339, 492
Hypnotism, recent studies of.....392
Hypodermics of Podophyllum.....22
Hypodermics of quinine.....35
Hypodermics in uterine hemorrhage.....165
Hysteria.....167, 203, 238
Hysteria under the influence of a magnet.....203

I.

Ice, home-made.....516
Ice cream, electrified.....471
Ice cream poison, electrical.....515
Ice, impure.....78
Ice water enemata in diarrhœa.....115
Impression, maternal.....273
Incandescent gas lamp.....339
Incontinence of urine.....25, 210
Indigestion.....123
Inebriety.....257
Infant, foreign body in the alimentary.....381
Infantile atrophy.....425
Infants, digestion of.....72
Infants, milk for.....210
Infection, surgical.....157
Inflammation, mammary.....474
Inflammation, pelvis.....142
Ingluvin.....27
Ingratitude.....300
Ingrowing toe-nail.....31, 117, 162
Inoculation against phthisis, preventive.....118
Inoculation, a new antirabies.....164
Insanity, an interesting case of.....472
Insanity, incipient.....488
Insects.....163
Insects as authors of epidemics.....120
Integrity, professional.....40
Intermittent fever.....110, 290
International Medical Congresss.....83, 256, 260, 344, 388, 390, 433, 434, 478
Intestinal obstructions.....288
Intubation of larynx.....325
Iodine from the skin, to remove stains of.....206
Iodoform, co lodion of.....240
Iodoform in Phthisis.....429
Iridectomies.....283
Iron in asthma, perchloride of iron.....161
Irrigating the ear.....309
Irritation, vesical.....369
Itch ointment.....211
Itching, linseed oil in.....293
Item.....26
Ivy Street Hospital.....37

J.

Jaborandi in eczema.....210
Jaundice.....36
Jones of New Orleans, professor.....213
Jones, Dr. Charles N.....227
Journals, support your home.....84

K.

Kendall, Dr. W. S.....475
Kidney amyloid degeneration of.....265
Kinnebrew, death of.....37
Kintz, Dr. J E.....127, 177, 213, 222
Knife, gastrotomy for removing a swallow'd.....63

L.

Labor, tartar emetic in.....463
Lactation and electricity.....373
Lamp, electric.....78
Lanolin in midwifery practice.....80
Lantanine as a quinine substitute.....381
Lantanine in malaria.....464
Laparatomy, a case of.....140
Laparatomy as an aid to Herniotomy.....22
Larynx, intubation of.....325
Laws against quackery.....40
Leffmann, Dr. Henry.....299
Lens, a new.....384
Letter from Germany.....66
Leucorrhœa, boracic acid in.....377, 506
Lick telescope, the.....119
Life, circulation not essential to.....162
Ligation of external iliac artery.....101
Ligation of femoral artery.....57

Lightning apparatus, exhibition of....339
Lightning rods....207
Lindney, Dr. W....108
Linseed oil in itching....293
Lister's latest antiseptic dressing....31
Lithuria....431
Localization of brain tumors....115
Locomotive, a gas....340
Los Angeles and vicinity, health of....108
Lumbago, instantaneous remedy for....517

M.

McKee, Dr. E. S....187
McMichael, death of Dr. C. ....475
McMichael, Dr. J. M....475
McRae, Dr. F. W....212
Maclay, Dr. C. B....523
Macon Medical Society....168
Magnet....340
Malaria, Lantanin in....464
Malarial fever....110
Malarial fever, hemorrhagic....213, 239, 277, 291, 390
Mammary abscess....116, 205, 426
Man an object of zoology....310, 357, 401, 441
Marriage by syphilitics, when safe....466
Maternal impressions....273
Measles....242
Medical Association, American....174, 302
Medical Association of Georgia...38, 87, 126, 174, 214
Medical Association of Georgia, to the....87
Medical Association of Stone Mount'n..258, 345
Medical Association, Ogeechee....124
Medical College, Atlanta....168, 388
Medical College, Southern....256, 388, 476
Medical Congress, International....83, 260, 344, 388, 433, 434, 478
Medical men to medical societies, duties of...45
Medical school for women in Russia....256
Medical Society, Anniston....168
Medical Society, Macon....168
Medical student, the tribulations of a....362
Medical students, tribute to....127
Medicinal eruptions....337
Medicine, how to avoid the taste of....4
Medicine, New York Academy of....146
Medicine, proper time to give....413
Medico, legal decision....344
Melon root....489
Menopause, Fowler's solution in....511
Menstruation, invariability of pulse in....193
Menthol, uses of....459
Mercury as a germicide, bichloride of....490
Mercury in diphtheria and carlatina, biniodide of....80
Mercury in scarlet fever....117
Message, an unique....257
Metals, rare....33
Meteor showers....119
Microbe of yellow fever....261
Micrococci of Vaccine....19
Micro-organism, detection....295
Micro-organism, the work of....230
Midwifery practice, lanolin....80
Military dogs....383
Milk for infants....210
Milk, poisoning by....18
Monilia, sputicola....412
Moore, Dr. M....311
Moore, surgeon-general....38
Morphia, succedanum of....514
Morphine, antipyrine as substitute for..510, 511
Morphine habit, new remedies for the....514
Morphine, solanine as a substitute for....69
Mortality in Europe....389
Mouth wash, antiseptic....430
Myrrh in infectious diseases....343

N

Nævus....137
Nares, inflammation of hair follicles in....165
Nasal catarrh, acute and chronic....36
Nasal Hypertrophy, scarification in....321
Navigation, aerial....33
Necrosis of Tibia....15
Needle in the arm....300
Nerve tonic in opium cases....385
Nervous phenomena from syphilitic pharyngeal ulceration....486
Nettle juice....382
Neuralgia....75, 200, 385
Neuralgia, electricity in....75
Neuralgia in pregnancy, dental....200
New York Academy of Medicine....146
Night sweats, agaricin for....425
Nitroglycerin....200, 416
Nullification, by an Eclectic....257

O.

Observations at the bedside....5
Obstetric section at the International Medical Congress....256
Obstetrics....5
Obstructions, intestinal....288
Ocular pains, ointment for....298
Ogeechee Medical Association....124
Oldham, Dr. John T....453
Ophthalmia neonatorum....30
Ophthalmia purulent....453
Ophthalmic practice, ergot in....512
Ophthalmology, some cases in....97
Opiates, advancing....388
Opium cases, nerve tonic in....385
Orme's address, Dr....389
Osborne, death of Dr. James....344
Otorrhœa, treatment of....508
Ovaries, be removed, when should the....498
Owen, death of Dr. W. G....475

P.

Palpitation....420
Paper, asbestos....252
Paracoto in dysentery....375
Paralysis in children, prognosis....368
Parson's local anæsthetic....430
Parts, union of severed parts....116
Pasteur and his new communication....415
Pasteur's hydrophobia inoculation....460
Patella, fracture of the....162, 424
Pelvic inflammation....142
Perforator and hook, a case requiring....141
Pernicious hemorrhagic fever....291
Pharyngitis....519
Pheno[illegible]ena from pharyngeal inflammat'n.486
Phillips, Dr. Latimer....268
Phlegmon, Resorcin inoculation in....381
Phosphorus in intermittent fever....290
Photographing the uterine cavity....292
Photographs, instantaneous....384
Photography, vest button....515
Phthisis, apparent recovery from....1 6
Phthisis, creasote in....462
Phthisis, glycerinein....298
Phthisis, iodoform in....429
Phthisis, bills for....167
Phthisis, preventive inoculation against....118
Phthisis, tonic in....81
Phthisis, the last treatment of....337
Physician, a young....380
Placenta prævia....280
Pleurisy, acute....32
Pneumonia....157, 184, 194
Pneumonia, antipyrine in....157
Podophylum, hypodermically....22
Poisoning by Fowler's solution....324
Poisoning by milk....18
Poisoning, cocaine....169
Poisons, a caution about selling....205
Porcher, Dr. F. P....104
Posology, for some new remedies....291
Potassium, iodide in diphtheria....290

Potassium, permanganate in Amenorrhœa..26
Powe l, Dr. T. S ... 83, 124, 434, 478
Pregnancy, bi-tartrate of potash in...162
Pregnancy, dental neuralgia and Headache 200
Pregnancy, ptyalism of...470
Professional integrity...41
Prolapse of rectum...382
Prurigo...517
Pruritus of the female genitals...473
Pruritus vulvæ...35, 211, 341, 343
Pseudo epilepsy of the retina, a case of... 11
Psoriasis... 254
Ptyalism of pregnancy...470
Puerperal convulsions...386
Puerperal fever...407
Pulmonary consumption contagious? is...494
Pulse in menstruation...193
Purulent ophthalmia...453
Pyridine in asthma...238
Pyrophosphate of iron, quinine and...79

Q.

Quackery, laws against...40
Queries...523
Query...522
Quigley, Dr...363
Quinine...6, 8, 35, 74, 213, 381, 402, 470, 514
Quinine, hypodermic solution of...35
Quinine in enceinte patients...6
Quinine in whooping cough... 74
Quinine, neutral hydrochloride of...470
Quinine, not good in hemorrhagic malarial 213
Quinine on the skin, rare effects of...8
Quinine substitute, lantanine as a...381
Quinine, when and how to give...402

R.

Readers, to our...521
Rebourgeon, Dr. C...261
Recovery from phthisis, apparent...116
Rectal surgery made easy...446
Rectum, prolapse of...382
Reeves, Dr J. R... 344
Remedies, posology of some new...291
Remittent fever...110
Renal calculus...155
Resection of tibia...15
Resorcin, inoculations in phlegmon...381
Respiration, method of inducing...469
Rest for the painful eyes...149
Restorers...122
Retina, pseudo-epilepsy of...11
Rheumatism...148, 201, 210, 254, 342, 421, 431
Rhus aromaticus...289
Riggs, Dr. F. C...11
Rorick's formula for Hemorrhoids...167
Rotheln, diagnosis of...244
Russia, medical school for women in...256

S.

Salicylate of ammonia...152
Salicylate of sodium...463
Salicylic acid...148
Salicylic acid and rheumatism...148
Santonin, danger of...243
Sarracenia flava in sporadic dysentery...288
Scabies...387, 518
Scarification in nasal hypertrophy...321
Scarlatina, biniodide of mercury in... 80, 117
Scarlet fever...80, 117, 182
Sciatica... 286, 338
Scoparin...206
Sea, a field of work under the...294
See, Professor Germain...501, 508
Shocks upon health, influence of...104
Singleton, death of Rev. D. P...168
Singultus...385
Sinkler, Dr. Wharton...147
Skeleton in the cupboard, the...472
Skin eruptions, chronic...81
Skin, rare effects of quinine on the...8
Sleep, positions that affect...503
Smith, Dr. E. C...368
Snakes...270
Snow...163
Society, Atlanta Medical...38
Sodium Salicylate...463
Solanine...69
Southern Medical College...37, 169, 256, 388, 476
Southern Med. College, commencement...169
Sparteine...206, 518
Spayed, she was not...22
Spleen pulp in anæmia...250
Stammering...324, 426
Steam, how Arago measured the power of...427
Stenocarpine a substitute for cocaine...467
Sterility of females, belladonna in...290
Stiles, Dr. C...128, 273
Stiles, Dr. S. W...310, 357, 401, 441
Stomach, cancer of...166
Stomach, dietary in catarrh of...412
Stone, address of Dr...433
Stone in the bladder...21
Stone Mountain Medical Association... 258, 345
Strangulated hernia...160
Stroud, Dr. H. E... 138, 309, 488, 490
Strychnia, arseniate of...196
Strychnine poisoning, charcoal an antidote to...510
Stye, treatment of...298
Styptic a good...35
Succedanum of morphia...514
Sueing to recover arreared subsciption...209
Sugar in the blood...251
Suprapubic cystotomy...71
Surgeon General of the U. S...38
Surgery, cocaine in minor...404
Surgery made easy, rectal...446
Surgical and Gynecological Association of Alabama...37
Surgical aphorisms...366
Suture, a painless...154
Syphilis... 167, 268, 474
Syphilis of middle ear, secondary...268
Syphilitics, marriage by... 466
Syringe the ear, how to...117

T.

Tait, Lawson...133
Takenaya, bark of...295
Taliaferro, Dr. V H...388, 493
Tannin, etheral solution of...32
Tartar emetic in labor... 463
Tartar from the teeth, to clean...122
Taste of Medicine, how to avoid the... 4
Teeth from a medico legal aspect, the...193
Telephone distances...78
Temperature, effect of carbolic acid on...509
Tendon, transplantation of...424
Tendons, treatment of ruptured and divided...507
Terebene...166
Tetanus at the International Medical Congress, remarks on...398
Tetanus cure, a case of traumatic...505
Thapsia plaster...293
Theine...201, 471
Theine, action of caffeine and...201
Threadworms in children...20
Throat, clergymen's sore...385
Thyroid gland, function of...318
Tibia, neurosis of...15
Tibia, resection of the entire...15
Tic douloureux, a case of...443
Tissue, waste of...519
Toe nail, ingrowing of...31, 117, 162
Tonic for children...81
Tonic in Phthisis...81
Tonsilitis...342
Toothache drops...293

Toothache, to stop ....253
Tran-fusion upon the heads of decapitated animals ....315
Tribulations of a medical student, the....363
Tributes to medical students....127
Trifolium compound in syphilis, syrup of...474
Tuberculosis....121, 141, 241, 377, 470
Tuberculosis, eucalyptol injections in....377
Tuberculosis, triple contagion of....241
Tumors, localization of brain....115
Tumors, Malignant....177, 222
Turpentine in croup....416
Typhoid fever....21, 118, 159, 204, 342, 458, 513
Typhoid fever, antipyrine in....159, 458
Typhoid fever, delirium of....342
Typhoid fever, ecchinoccea for....118
Typhoid fever from a single dose....204
Typho malarial fever....390
Tyson, Dr. James....265

U.

Ulceration, nervous phenomena from pharyngeal ....486
Ulcerations....241, 247, 248
Ulcerations, the carbolized spray in.... 248
Ulcerations, the subiodide of bismuth in...241
Ulcers, chronic ....247
Ulcus cruris....465
Union of severed parts....116
Urethritis, modern treatment of....417
Urine, incontinence of....25, 211
Uterine cavity, photographing the....292
Uterine hemorrhage....165, 211
Uterus, dilating the....197
Uterine, Indications and rationale in washing the puerperal....502
Uterus, on vaginal extirpation of....75

V.

Vaccinating, directions for....378
Vaccine micrococci ....19
Vance, Dr. A P. Morgan....404
Vascular diseases, barium chloride for....473
Vegetable, meat eaters....208
Vertigo .... 20
Vesical calculi.... 328
Vesical irritation....369
Vest button photography....515
Veterinary medicine....379
Vomiting....32, 77
Vomiting, certain forms of....336
Vomiting, uncontrollable....32
Vomiting of cholera infantum....387

W.

Wadsworth, death of Dr. J. N....388
Wall paper, arsenic in....468
Wall paper, simple test for....295
Warts, a cure for....206
Wash, antiseptic, mouth....430
Wasp, a wise....427
Waste of tissue....519
Water, diet....25
Water for babies.... 334
Water in eye lotions distilled....287
Webber, Dr. S. G....13
Weight, to increase....229
Weight to reduce....226
Westmoreland, Dr. Robert W....212
Wethington, Dr....520
What about chloroform ?....7
What about forceps ?....7
What about ghosts ?....120
Whitlow ....423, 469
Who is he ?....217
Whooping cough, quinine in...74, 79, 165, 243, 509
Wile, death of Dr. Henry....212
Wile, the case against Dr. Henry....169
Wilkinson, Dr. C. H....8, 410
Wine, a laxative and tonic.... 254
Wine in bed wetting, red....458
Word, Professor R. C....261, 388
Work under the sea, a field of....294
Worms in children....20
Worms, intestinal....343
Wounds, aseptic dressing of punctured....359
Write, our friends must....520
Wright, Dr. J. C....19

Y.

Yellow fever....249, 262, 274
Yellow fever, inoculation and theory of....274
Yellow fever, preventive inoculation of....249

Z.

Zoology, man an object of....3 0, 357, 401, 441

### RECEIPTED.

For 1886—Drs. W. B. Maxwell, W. L. Wright, A. H. Sellers, H. S. Bruce; J. E. Gilchreest, J. A. Ryan, Thomas Bailey, to July; F. H. Johnson, S. G. Dilburn, E. S. Foster, T. L. Appleby, Z. T. Young, T. J. Brasher, John H. Henry.

For 1887—Drs R. C. McCann, T. P. Olliver. J. E. Frippe, P. R. Cortelyou, Jonathan Jones, Robert T. McGill, John L. Waldrop, Evan Johnson, W. R. Smyth, W. W. Pelham, T. R. Smoot, J. S. Timmons, William A. Brown, M. Gilsy, S M. Hogan.

For 1888—Dr. J. P. Alred.

